# *In vivo* targeting of a variant causing vanishing white matter using CRISPR/Cas9

**DOI:** 10.1016/j.omtm.2022.02.006

**Published:** 2022-02-23

**Authors:** Anne E.J. Hillen, Martina Hruzova, Tanja Rothgangl, Marjolein Breur, Marianna Bugiani, Marjo S. van der Knaap, Gerald Schwank, Vivi M. Heine

**Affiliations:** 1 Department of Pediatrics and Child Neurology, Emma Children's Hospital, Amsterdam Neuroscience, Amsterdam UMC, De Boelelaan 1117, 1081 Amsterdam, the Netherlands; 2 Department of Biology, Institute for Molecular Health Sciences, ETH Zurich, Otto-Stern-Weg 7, 8093 Zurich, Switzerland; 3 Department of Functional Genomics, Center for Neurogenomics and Cognitive Research, Amsterdam Neuroscience, VU University, De Boelelaan 1085, 1081 Amsterdam, the Netherlands; 4 Institute of Pharmacology and Toxicology, University of Zurich, Winterthurerstrasse 190, 8057 Zurich, Switzerland; 5 Department of Complex Trait Genetics, Center for Neurogenomics and Cognitive Research, Amsterdam Neuroscience, VU University, De Boelelaan 1085, 1081 Amsterdam, the Netherlands; 6 Department of Child and Adolescence Psychiatry, Emma Children's Hospital, Amsterdam Neuroscience, Amsterdam UMC, De Boelelaan 1085, 1081 Amsterdam, the Netherlands

**Keywords:** vanishing white matter, leukodystrophy, CRISPR/Cas9, gene therapy, gene editing

## Abstract

Vanishing white matter (VWM) is a leukodystrophy caused by recessive variants in subunits of eIF2B. At present, no curative treatment is available and patients often die at young age. Due to its monogenic nature, VWM is a promising candidate for the development of CRISPR/Cas9-mediated gene therapy. Here we tested a dual-AAV approach in VWM mice encoding CRISPR/Cas9 and a DNA donor template to correct a pathogenic variant in *Eif2b5*. We performed sequencing analysis to assess gene correction rates and examined effects on the VWM phenotype, including motor behavior. Sequence analysis demonstrated that over 90% of CRISPR/Cas9-induced edits at the targeted locus are insertion or deletion (indel) mutations, rather than precise corrections from the DNA donor template by homology-directed repair. Around half of the CRISPR/Cas9-treated animals died prematurely. VWM mice showed no improvement in motor skills, weight, or neurological scores at 7 months of age, and CRISPR/Cas9-treated controls displayed an induced VWM phenotype. In conclusion, CRISPR/Cas9-induced DNA double-strand breaks (DSBs) at the *Eif2b5* locus did not lead to sufficient correction of the VWM variant. Moreover, indel formation in *Eif2b5* induced an exacerbated VWM phenotype. Therefore, DSB-independent strategies like base- or prime editing might better suited for VWM correction.

## Graphical abstract

**Figure:**
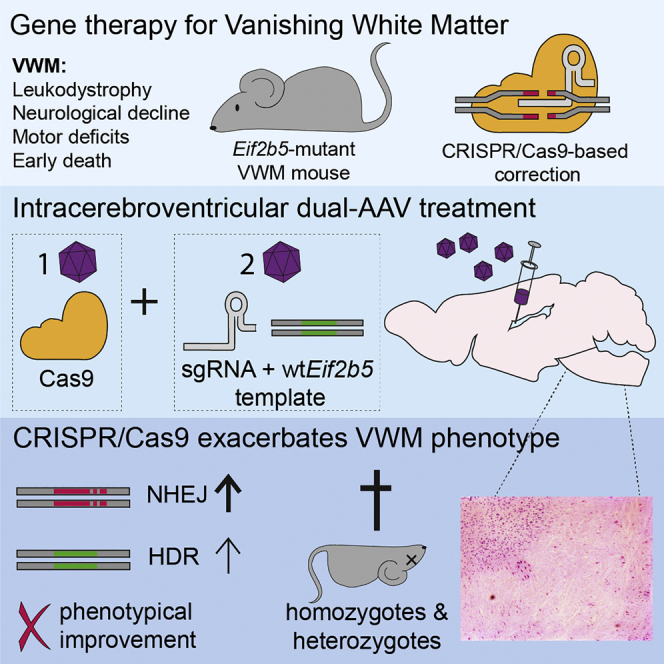

## Teaser

Vanishing white matter (VWM) is a devastating leukodystrophy caused by bi-allelic variants in genes related to mRNA translation. No curative treatment is available and patients often die at young age. We tested CRISPR/Cas9-mediated gene therapy using dual AAV. However, low correction rates and indel formation lead to exacerbated VWM phenotypes.

## Introduction

The CRISPR/Cas9 nuclease system is a robust genome editing platform with great potential for applications in biomedical research and the clinic.^1^ It consists of a Cas9 endonuclease, which is targeted to the genome via a programmable single-guide (sg)RNA. In mammalian cells, Cas9-generated DNA double-stranded breaks (DSBs) can induce insertion or deletion (indel) mutations through non-homologous end joining (NHEJ), thereby creating frameshifts and gene knockouts, or correct a variant via homology-directed repair (HDR) using a DNA donor template. While high efficiency of the CRISPR/Cas9 technology in generating indel mutations has stimulated the development of gene therapies for diseases where loss of a gene function is desired,^2^^,^^3^ low rates of HDR in most mammalian cell types have so far hampered broad application of this system for recessive genetic disorders.

Here, we aim to employ CRISPR/Cas9 for the treatment of the monogenic neurodegenerative disease vanishing white matter (VWM; OMIM #603896). VWM is one of the most prevalent leukodystrophies,^4^ and is caused by pathogenic variants in the genes encoding the eIF2B subunits (*EIF2B1*–*EIF2B5*). The disease is characterized by chronic and episodic neurological decline, including motor deficits, and can result in early death.^5^ No curative treatment is currently available.^5^ As a guanine exchange factor, eIF2B forms a rate-limiting factor for mRNA translation into protein; indeed, pathogenic variants in eIF2B decrease translation rate.^6^ Previous studies have shown that astrocytes drive VWM pathology via dysregulated activity of the integrated stress response (ISR), in which eIF2B is a key component.^7^^,^^8^ One-time early intervention as a treatment for VWM has been illustrated previously in a mouse model containing a bi-allelic variant in *Eif2b5*, by transplanting healthy glial cells into newborn pups. However, only partial improvement in the VWM phenotype was observed.^9^ Here, we attempted to repair the pathogenic variant of this VWM mouse model *in vivo* in the brains of newborn pups via CRISPR/Cas9, to assess if this technology can be applied to alleviate the VWM disease phenotype.

## Results

### Intracerebroventricular administration of CRISPR/Cas9

It is thought that astrocytes drives VWM pathology.^10^ We therefore first investigated the glial tropism of the Adeno-associated virus (AAV) serotypes AAV2/5, AAV2/8, and AAV2/9, each expressing GFP under a cytomegalovirus (CMV) promoter. Intracerebroventricular (ICV) injections of each serotype (Table S1) resulted in GFP expression throughout the brain (Figures S1A–S1E). However, quantification showed that AAV2/8 transduced the highest number of astrocytes (42.7%; Figure S1G), and also showed the highest preferential transduction of cells in the white matter (Figure S1F, white matter tracts), prompting us to continue with AAV2/8 for subsequent CRISPR/Cas9 experiments.

**Figure 1:**
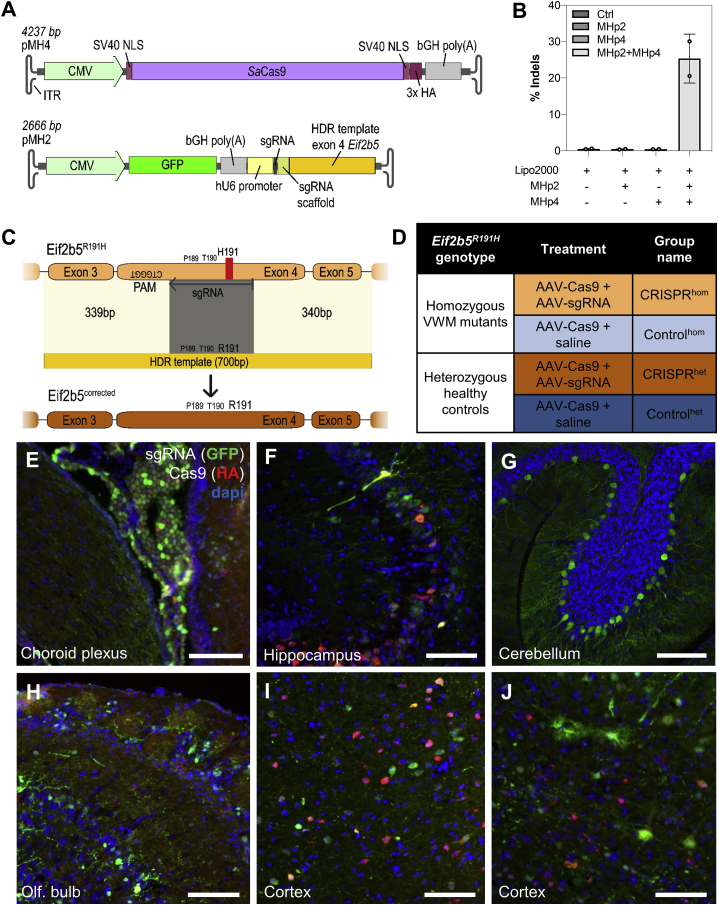
Overview of the CRISPR/Cas9 system, experimental setup, and transduction patterns *in vivo* (A) Schematic overview of the dual AAV constructs. (B) *In vitro* testing of the sgRNA. HEK293T reporter cells were transfected with the respective treatment using Lipofectamine 2000 (Lipo2000). Insertions and deletion (indel) frequency was determined by next-generation targeted amplicon sequencing. Values represent mean ± SD of two technical replicates. (C) Graphical illustration of the used HDR design. The *Eif2b5*^*R191H*^ disease-causing mutation (red) on exon 4 was targeted by a 21 bp long sgRNA (gray) to induce a double-stranded DNA (dsDNA) break. The disease-causing mutation lies in the center of the 700 bp long HDR template (orange). The HDR template with homology-arms of 349 bp and 350 bp corrects the R191H mutation and additionally introduces a silent mutation in the sgRNA seed region. (D) Overview of treatment groups for *in vivo* studies (*n* = 79 per group). Cells expressing Cas9 (tagged with HA, red) and/or sgRNA and DNA donor template (tagged with GFP, green) in (E) the choroid plexus, (F) hippocampus, (G) cerebellum, (H) olfactory bulb, (I) deeper cortical layers, and (J) superficial layers of the cortex of animals between 5 and 9 weeks of age. Scale bars represent 75 μm.

To correct the pathogenic *Eif2b5*^*R191H*^ variant in a VWM mouse model, we generated a *Sa*Cas9 system that targets a CTGGGT PAM site 11 base pairs (bp) upstream of the R191H variant, generating a DSB in close proximity (8 bp) to the variant. Two constructs were generated (Figures 1A and 1C): one expressing a hemagglutinin (HA)-tagged *Sa*Cas9 under the CMV promoter, and another encoding for the 700 bp donor template; a GFP expressed under the CMV promoter; and the sgRNA under the U6 promoter. In HEK293T cells with the *Eif2b5*^*R191H*^ locus stably integrated, the *Sa*Cas9 system led to ∼25% indel formation upon transfection with Lipofectamine (Figure 1B). To test the efficacy of CRISPR/Cas9-mediated correction of the VWM variant *in vivo*, we injected the two AAVs together to jointly transcribe a functional CRISPR/Cas9 complex (referred to as CRISPR, *n* = 79) in the lateral ventricles of neonatal mice. A control group (referred to as control) was injected with only the Cas9-encoding AAV and saline (Figure 1D, *n* = 79). As expected from previous studies^11^ and our pilot experiments (Figure S1), ICV injection showed transduction of cells throughout the brain (Figures 1E–1J).

### CRISPR/Cas9-treated mice show indel formation and low HDR-based correction

**Figure 2:**
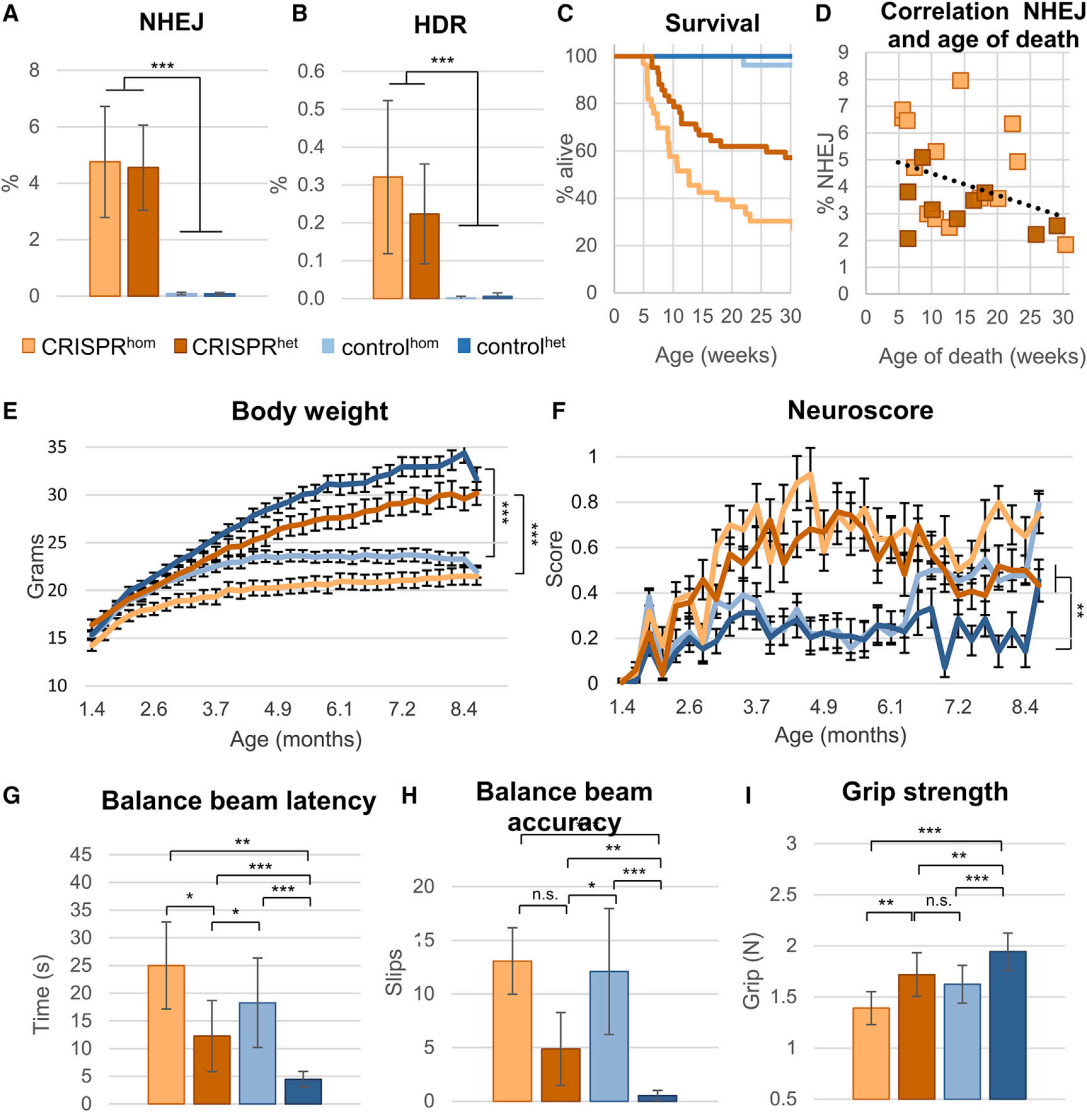
The CRISPR/Cas9-induced indel to correction ratio exacerbates the VWM phenotype (A) Next-generation sequencing (NGS) was used to assess the percentage of NHEJ edits per treatment (statistics by independent-samples t test. CRISPR n = 51, control n = 46). (B) The percentage of HDR edits per treatment, as determined by NGS (statistics by Whitney-Mann U test. CRISPR n = 51, control n = 46). (C) Survival curve of animals split into genotypes and treatment groups. (D) The correlation between age of death and percentage of NHEJ. (E) Development of body weight and (F) Neuroscore of CRISPR and control animals throughout development (CRISPR hom n = 30, het n = 42; control hom n = 40, het n = 37). (G) Speed and (H) accuracy of traversing a balance beam, as well as (I) grip strength, were assessed in 7-month-old animals (CRISPR hom n = 10, het n = 24; control hom n = 21, het n = 25). A Kruskal-Wallis test was used for (E) and (H). A Welch test with Games-Howell *post hoc* was used for (G). A one-way ANOVA with Tukey *post hoc* was used for (I) ∗p < .05; ∗∗p < .01; ∗∗∗p < .001. Values are represented as the mean ± SD

To discriminate between indel formation and precise correction of the R191H variant by HDR, next generation sequencing (NGS) was performed on amplicons generated by PCR on genomic DNA isolated from brain lysates. To avoid amplification of randomly integrated DNA donor templates, a primer pair binding outside of the HDR template sequence was used. A significantly higher percentage of both NHEJ (T(50.1) = 19.1, p < .001; Figure 2A) and HDR (U = 8.0, p < .001, *r* = .877; Figure 2B) was observed in the CRISPR groups (n = 51) compared with the control groups (n = 46) in both diseased *Eif2b5*^*hom*^ and healthy *Eif2b5*^*het*^ animals (see Table S1). However, more NHEJ than HDR was observed in CRISPR-treated animals (Z = −6.2, p < .001, *r* = .870), with an average HDR rate (M = 0.27% ± 0.17%, n = 51) roughly 17 times lower than that of NHEJ (M = 4.64% ± 1.7%, n = 51). These results suggest that even if the activity of CRISPR/Cas9 nucleases could be increased, e.g., by using higher viral titers or stronger promoters, correction efficiency would remain low due to the majority of edits at the locus being indel mutations.

### Premature death in CRISPR/Cas-treated mice

Starting from 5 weeks after treatment, CRISPR animals experienced unexpected premature death (Figure 2C), showing sudden and rapid deterioration. All animals received a daily visual checkup to assess hunched posture, grimaces, piloerection, poor grooming, social isolation, movement/activity levels, and motor skills (part of the VWM phenotype). Before the unexpected premature deaths, animals showed no signs of discomfort or weight loss. A veterinarian inspected three animals that experienced sudden deaths and observed no gross internal aberrations. CRISPR^hom^ and CRISPR^het^ mice were equally affected (n = 23, and n = 21, respectively) and did not differ in the average age of death. Since no outward phenotype was apparent in the control group receiving AAV-Cas9 alone, it is unlikely that the deterioration seen in the CRISPR animals was due to Cas9 expression.

As the animals aged, the number of deaths decreased (Figure 2D, hom n = 17, het n = 9). Only one animal of the control group, which solely expressed *Sa*Cas9 and no sgRNA, died without clear cause (Figure 2C). This indicates that the occurrence of the unexpected sudden deaths was specifically associated with functional CRISPR/Cas9 complexes, and the generation of DSBs and indel mutations in *Eif2b5*. To test if indel rates correlated with the occurrence of unexpected deterioration, we plotted the percentage of NHEJ to the age of death in the CRISPR group. Age of death did not differ between CRISPR^hom^ (n = 21) and CRISPR^het^ (n = 30) animals, nor did the percentages of either NHEJ (Figure 2A) or HDR (Figure 2B; Table S1). Moreover, in CRISPR mice that died spontaneously, a statistical trend was seen in the negative correlation between NHEJ and age of death (r_s_(23) = -.401, p = .058; Figure 2D). This was not observed in the control group, suggestive of a deleterious effect of indel formation in the *Eif2b5* locus.

### CRISPR/Cas9 treatment worsens performance on VWM phenotyping assays

To inspect whether indel mutations in CRISPR animals affected VWM severity, we assessed various aspects of the VWM phenotype, such as body weight and motor skills. CRISPR^hom^ and CRISPR^het^ animals maintained a slightly lower weight than their control group counterparts throughout development (Figure 2E; Table S1). Moreover, CRISPR^hom^ mice (n = 30) weighed significantly less than CRISPR^het^ (n = 42) mice at the first weighing (42 days of age; H(3) = 9.178, p < .001). This weight difference between genotypes was not observed in the control groups until later in life (H(3) = 35.991, p < .001 at 7 months of age, control^hom^ n = 21, control^het^ n = 25), as expected from the VWM phenotype. The Neuroscore of both CRISPR^hom^ and CRISPR^het^ animals was worsened by CRISPR/Cas9 treatment compared with control^het^ animals (assessed at 7 months; H(3) = 24.936, p < .005) and was matched by control^hom^ animals only later in life, when this latter group started to deteriorate due to the VWM genotype (Figure 2F; Table S1). In CRISPR groups (hom n = 10, het n = 25), the motor skills that become increasingly perturbed in VWM displayed worse speed (F(3,25.478) = 40.484, p < .005) and accuracy (H(3) = 53.796; p < .005) while traversing a balance beam (Figures 2G and 2H), and poorer grip strength (F(3,76) = 18.525, p < .005; Figure 2I) compared with the control groups (hom n = 21, het n = 24). The performance of CRISPR^hom^ mice was not improved compared with either of the control groups. In addition, CRISPR^het^ animals performed significantly worse in every motor assay compared with control^het^ animals. These data strongly suggest that CRISPR/Cas9-induced indel mutations in *Eif2b5* exaggerated the VWM phenotype.

### CRISPR/Cas9 induces severe VWM-related pathology in the brainstem

**Figure 3:**
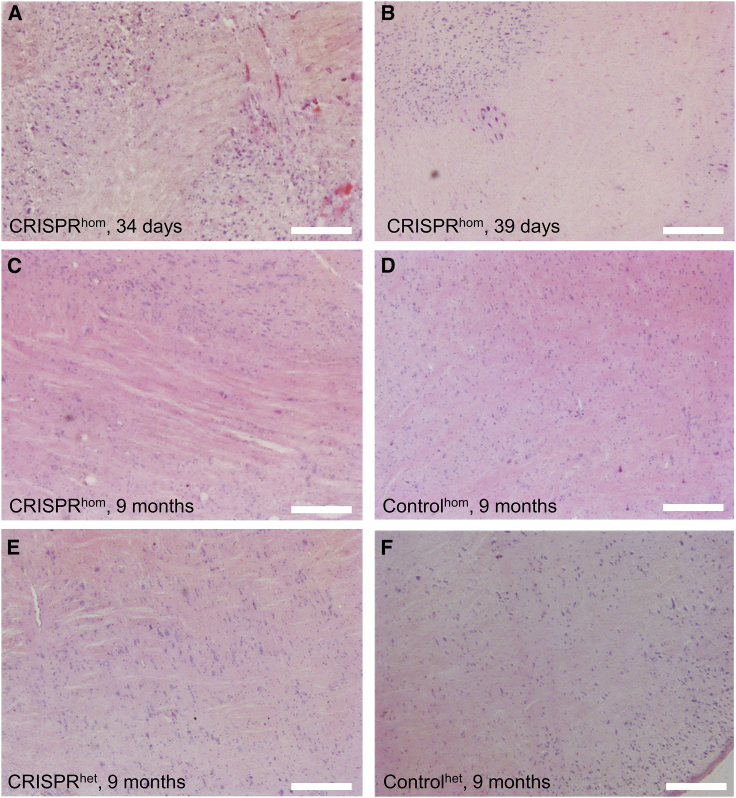
CRISPR/Cas9-treated animals show necrosis in the brainstem (A and B) CRISPR animals that deteriorated rapidly and died around 5 weeks of age showed necrotic tissue in the brainstem and hypoxic neurons, as shown in H&E analysis. Necrosis was also observed in 9-month-old (C) CRISPR^hom^ or (E) CRISPR^het^ animals, but was not present in age-matched controls of either genotype: hom (D), het (F). n = 4 early death CRISPR^hom^ animals; n = 1 early death CRISPR^het^ animal; n = 1 for CRISPR^hom^, CRISPR^het^, and control^het^ animals of 9 months old; n = 2 for control^hom^ animals of 9 months old. Scale bars represent 100 μm.

To assess pathological effects of CRISPR/Cas9 treatment, histological inspections were performed on hematoxylin & eosin Y (H&E)-stained brain sections. We observed necrotic tissue in the brainstem of CRISPR mice (unexpected deaths CRISPR^hom^ n = 4, het n = 1; Table S1; Figures 3A–3C, and 3E), including the intrinsic nuclei and reticular formation, qualifying as coagulative necrosis with decrease to loss of nuclear basophylia. Necrosis was acute, because it was not accompanied by reactive gliosis, accruing of macrophages, or neovascularization. Indeed, the lack of inflammation may also be explained by the acute nature of the lesions. Hypoxic red neurons were observed throughout the brain. Brainstem deterioration was only observed in CRISPR/Cas9-treated animals, and not in control animals (Figures 3D and 3F). Thus, CRISPR/Cas9 treatment induces a phenotypic component of VWM^12^^,^^13^ that likely led to an exacerbated deterioration of mice.

## Discussion

We harbored the CRISPR/Cas9 nuclease system in the attempt to correct the causative variant in *Eif2b5*^*R191H*^ VWM mice. We report a high death rate in the CRISPR/Cas9-treated group and an enhanced VWM phenotype. Our data therefore suggest a strong contra-indication for the use of Cas9-mediated gene editing in VWM. NGS data of the CRISPR/Cas9-treated mice show a 17-fold higher rate of indel formation compared with HDR-mediated gene correction. We therefore conclude that the likely further disruption of the remaining housekeeping function of the *Eif2b5*^*R191H*^ allele leads to incompatibility with life. Previous studies have developed approaches to enhance HDR efficiency *in vitro* in cell lines. These include NHEJ inhibition *in vitro*,^14^, ^15^, ^16^, ^17^ synchronization of the cell cycle,^18^, ^19^, ^20^ modifying DNA template length^21^, ^22^, ^23^ and (a)symmetry,^24^, ^25^, ^26^ and conjugating the repair template to the Cas9 ribonucleoprotein complexes.^27^ It would be interesting for future endeavors to assess whether these approaches could also be adapted to improve HDR efficiency in the brain *in vivo*, in order to reduce the formation of deleterious indels. Moreover, assessment of HDR/indel at other targeted loci would allow us to make more generalizable statements on the precision of *in vivo* genome editing in the brain using CRISPR/Cas9 nucleases.

The sharp decline in viability of our CRISPR/Cas9-treated mice started at 5 weeks of age. In mice, myelination starts in the spinal cord at birth and increases rapidly until at least P45.^28^^,^^29^ If indels have formed in *Eif2b5*, eIF2B activity presumably drops sharply and thus limits the rate of protein synthesis. This may have created a metabolic load that surpasses the threshold at which perturbed eIF2B function becomes non-viable, especially during metabolically demanding times such as development. As time progresses, the death rate decreased, possibly because metabolism in the central nervous system (CNS) becomes more lax during adulthood.^30^^,^^31^ Additionally, severe pathology was found in the brainstems of CRISPR/Cas9-treated mice, showing acute necrosis. The location of these necrotic changes in the brainstem helps explain the rapid deterioration of CRISPR/Cas9-treated mice. Considering the indispensable role of eIF2B in healthy development and in VWM disease severity, indel formation without sufficient correction is likely resulting in premature death at young age.

Surviving animals did not show increased correction compared with animals that died prematurely. Instead, *Eif2b5*^*hom*^ VWM animals treated with CRISPR/Cas9 were unimproved on a series of phenotypic assays compared with *Eif2b5*^*hom*^ animals in the control group. Moreover, indel formation following CRISPR/Cas9 treatment induced a VWM phenotype in normally healthy *Eif2b5*^*het*^ animals: these animals displayed worsened Neuroscores and poorer performance on motor assays when compared with *Eif2b5*^*het*^ animals that only received the Cas9-encoding vector. Moreover, involvement of the brainstem was observed in CRISPR/Cas9-treated animals, which is also part of the VWM phenotype. Involvement of the brainstem has been observed in severe patients during rapid decline.^12^^,^^13^ While the R191H mutation by itself has not been able to recapitulate this aspect of human VWM disease in the VWM mouse phenotype, VWM-associated brain stem involvement has likely been induced in the CRISPR/Cas-treated VWM mice by additional mutation of *Eif2b5* by indel formation. While co-localization of a necrosis marker with Cas9 and sgRNA would be required to directly attribute the necrosis to CRISPR/Cas9-mediated gene edits, our data suggest that CRISPR/Cas9 treatment resulted in a more severe VWM phenotype, including necrosis in the brain stem.

Together, our data show that the standard CRISPR/Cas9 nuclease technology could not be used to efficiently correct pathological *Eif2b5* alleles. Despite AAV transduction rates and editing rates comparable with a previous study using ICV injection of AAV-encoding CRISPR-Cas9,^32^ the vast majority of edits were indel mutations that resulted in lethality in treated mice, likely due to the housekeeping function of eIF2B. A second “hit” following indel formation, such as increased metabolic load during development or stressors, could cause the CRISPR-induced exacerbation of VWM variants to result in rapid decline. We speculate that base editors or prime editors, which are more precise than nucleases and enable gene correction without DSB formation and HDR, might be better suited for the correction and treatment of VWM.

## Materials and methods

### Experimental animals

All mouse experiments conducted in this study were approved by the Animal Approval Committee of the VU University Amsterdam and were carried out following guidelines of the Dutch government. A well-characterized mouse model for VWM^33^ with an induced point mutation at *Eif2b5*^*R191H*^ was used to assess the effects of CRISPR/Cas9 treatment on the VWM phenotype. These mice have a C57Bl/6J and *Rag2*^null^ background. Homozygous offspring (Eif2b5^hom^) develop a VWM phenotype, whereas heterozygous littermates do not and were used as healthy controls (Eif2b5^het^).

Every individual mouse was considered an experimental unit. An *a priori* power analysis was performed based on previously quantified *Eif2b5*^*R191H*^ phenotypic effects (on body weight, grip strength, latency, accuracy; Dooves et al.^33^). Effect sizes were used to calculate a β of .80 and suggested that n = (6, 12, 4, 9, corresponding to the assays abovementioned) 4–12 animals of each genotype per group were required. As we anticipated the effect of editing to be less pronounced than genotype-mediated effects, we aimed for the higher end of this range. We injected n = 79 neonates with AAV-sgRNA + AAV-Cas9 and n = 79 animals with AAV-Cas9 + saline. Due to the setup of the breeding, offspring had a 50% chance to be homozygote mutant, thus resulting in groups of ∼n = 40 per genotype (VWM versus control) per condition (CRISPR/Cas9 versus Cas9-saline control). Divided over three planned time points (which were ultimately precluded by the premature deaths), this would have resulted in 13 or 14 animals per group. Animals were excluded from analysis only if the obtained samples (tissue, DNA) were of insufficient quality for proper processing, e.g., due to delayed collection after premature death.

### CRISPR construct design

Vector pMH2_eGFP_sgRNA-EIF2B5 and vector pMH4_SaCas9 (cloned from Addgene plasmid #61591, a gift from Feng Zhang) were used to produce AAV2/8 virus for ICV injections into homozygous (hom) and heterozygous (het) *Eif2b5*^*R191H*^ neonates. The experimental group receiving CRISPR/Cas9 treatment (CRISPR) were injected with a 1:1 mix of the virus encoding Cas9 (pMH4) together with the virus encoding the sgRNA, the DNA donor template to correct the R191H variant, and a GFP tag (pMH2). The genomic target site of the 700 bp HDR template was sequenced in the mice we used in this study to exclude the existence of mismatches in the HDR arms. The control group received injections of virus encoding for Cas9 mixed with saline.

For pMH2 (eGFP-sgRNA-*Eif2b5*) *Sa*Cas9 on an AAV backbone containing AAV2 ITRs from Addgene plasmid #61591 (a gift from Feng Zhang) has been replaced by eGFP using AgeI and KpnI, and the spacer for targeting *Eif2b5*^*R191H*^ (CATGGCAGTGTGTAGGGTGG) has been cloned between the U6 promoter and sgRNA scaffold using BsaI digest. The DNA donor template comprised the *Eif2b5* locus sequence 350 bp upstream and downstream of the R191H variant, and was cloned into the plasmid downstream of the sgRNA scaffold using NotI digest. The donor template contained an additional silent T to A mutation five bases upstream of the R191H variant to exclude cutting of the donor template by the sgRNA. For pMH4 (*Sa*Cas9), the U6 promotor and the sgRNA scaffold from Addgene plasmid #61591 were removed and replaced by a small multiple cloning site using a KpnI and NotI digest.

### *In vitro* testing of sgRNA efficiency

Cutting efficiency of the sgRNA was assessed *in vitro* in a HEK293T reporter cell line. To generate the piggyBac disease reporter plasmid, inserts with homology overhangs for cloning were ordered from Integrated DNA Technologies (IDT) and cloned into the pPB-Zeocin backbone using HiFi DNA assembly MasterMix (New England Biolabs [NEB]). The piggyBac disease reporter plasmids and helper plasmid were transfected using Lipofectamine 2000 according to the manufacturer's instructions. For sgRNA testing, pMH2 and pMH4 were co-transfected at a 1:1 ratio using Lipofectamine 2000 according to the manufacturer's instructions. DNA was isolated using direct lysis buffer (10  μL of 4× lysis buffer [10 mM Tris-HCl pH 8, 2% Triton X-100, 1 mM EDTA, 1% freshly added proteinase K]) and incubated at 60°C for 60 min and 95°C for 10 min. Targeted amplicon sequencing was performed as described below using the following forward primer CTTTCCCTACACGACGCTCTTCCGATCTNNNNNNGATCACAGAGTTGGGCAGAC and reverse primer GGAGTTCAGACGTGTGCTCTTCCGATCTNNNNNNCACTTCCAGAAGACCCAAGG.

### Virus production

All pseudotyped AAV8 vectors (AAV2/8) were produced by the Viral Vector Facility of the Neuroscience Center Zurich. AAV vectors were ultracentrifuged and diafiltered. Physical titers (vector genomes mL^−1^) were determined using a Qubit 3.0 Fluorometer. In brief, the Qubit Fluorometer 3.0 (Life Technologies) was used to measure the concentrations (ng/mL) of the extracted genomes by denaturation at 95°C for 5 min, after which the readings were converted to genome copies (gc)/μL using the genome's molecular mass and Avogadro's constant. Identity of the packaged genomes of each AAV vector was confirmed by Sanger DNA sequencing by Mycrosynth AG (Balgach, Switzerland), testing 500 ng of denatured AAV using the sequencing primer CGCAAATGGGCGGTAGGCGTG. AAV2/8 viruses were stored at −80°C until use.

### ICV injections

Mice were injected within 24 h of birth. The instrumental setup and handling of pups was as described by Dooves et al.^9^ In short, half of the pups of a litter were collected and placed on a heat mat until injected. Upon injection, pups were anesthetized on ice for 7 min and placed on an ice-cold platform. The collected half of the litter were placed back with the mother when all pups had regained consciousness. The injection protocol was adapted from Kim et al.^34^ Viruses were thawed on ice and mixed to an equimolar (1:1) concentration. For the experimental group receiving CRISPR/Cas9 treatment, 1.05E^11^ vg in 3 μL was injected per lateral ventricle.^35^, ^36^, ^37^, ^38^ For the control group, AAV-Cas9 virus was mixed with saline. A microinfusion pump controlled a 10-μL syringe (Hamilton) with 34G beveled needle (World Precision Instruments) in a stereotaxic instrument to slowly inject^9^ in predetermined coordinates (millimeters from Lambda: X = ±0.8, Y = ±1.5, Z = −1.7, −1.5) as described.^34^ The needle was left in place for 1 min before slowly retracting over the course of 30 seconds. It should be noted that coordinates were adjusted slightly according to size differences among pups whenever deemed necessary. Separate needles were used for the AAV-sgRNA + AAV-Cas9 mix for the experimental group (CRISPR) and the saline + AAV-Cas9 mix for the control group (control). All pups of a litter received the same construct(s). Administration of constructs was not randomized across litters.

### Processing of tissue

Animals were culled by administration of an overdose of Avertin (12.5 g/mL 2,2,2-tribromoethanol in tertiary amyl alcohol) if deteriorating rapidly or at planned time points of 12 weeks of age or 5, 8, or 9 months of age (Table S1). When unresponsive, transcardial perfusion was performed with PBS followed by 4% PFA. Skulls and spinal columns were incubated in 4% PFA overnight at 4°C with agitation before isolation of the brain and spinal cord, respectively. Tissues were then incubated overnight in 30% sucrose (w/v in PBS) at 4°C with agitation until saturated. The hemispheres were separated. Spinal columns were horizontally cut into a cervical, thoracic, and lumbar section. Tissue was embedded in optimal cutting temperature (OCT) compound and snap frozen in a dry ice and 2-methylbutane bath. Embedded tissue was stored at −80°C.

### NGS

NGS was performed on brain lysates to assess the percentages of NHEJ and HDR in edited cells. DNA was isolated with UltraPure phenol:chloroform:isoamyl alcohol (Thermo Fisher) according to the manufacturers' instructions. Briefly, lysates were spun down at 13,300 RPM for 5 min to pellet the lysate. Supernatant was pipetted into a new Eppendorf tube and phenol:chloroform:isoamyl alcohol was added to lysates 1:1. Samples were vortexed vigorously for at least 20 sec before spinning down for 15 min at 13,300 RPM. After successful phase separation, the aqueous top layer supernatant was pipetted into a clean Eppendorf with care not to disturb the protein or lipid layers. Half the amount of supernatant volume of 3 M NaAc pH 4.5 was added, as well as twice the volume of 100% EtOH.

The region of interest within the *Eif2b5* locus was amplified from 100 ng of genomic DNA using the primer pair (5′-CCAGGAAAGCCACAGAGGAG-3′ and 5′-CCCTAGATTTGGTTCCCAGCA-3′) in a 50-μL PCR reaction using the NEBNext High-Fidelity 2× PCR Master Mix (M0541L, NEB), annealing at 64°C, for 30–35 cycles. The PCR product was purified with magnetic beads (Sera-Mag Select, GE Life Science) and used as a template for a second PCR with primer pair 2 (5′-CTTTCCCTACACGACGCTCTTCCGATCTNNNNNNGACTAACTGTGCCTCTGGTTCT-3′ and 5′-GGAGTTCAGACGTGTGCTCTTCCGATCTNNNNNNGGAAGTGAAGAACCCTGTTGG-3′). In the second PCR, a smaller DNA region within the first PCR product was amplified and Illumina adaptors were added in a 25-μL PCR reaction using NEBNext High-Fidelity 2x PCR Master Mix (M0541L, NEB), annealing at 55°C, for eight cycles. The PCR product from the second PCR was again purified with magnetic beads and used as a template for a third PCR reaction. In the third PCR reaction, index barcodes were added to each sample in a 25-μL PCR reaction using NEBNext High-Fidelity 2x PCR Master Mix (M0541L, NEB), annealing at 55°C, for eight cycles. PCR products were validated using 2% agarose gels. Amplified libraries were pooled, gel purified, and normalized to approximately equal molar ratio using a Qubit 3.0 fluorometer. Samples were sequenced using the MiSeq Micro v2 Illumina or the MiSeq Nano v2 Illumina kit (150 bp, paired-end reads). Results were analyzed with CRISPResso2.^39^

### Motor skill testing

Animals were tested on various VWM phenotypes, including bodyweight, Neuroscores, and motor skills. Assessments of weight and Neuroscore were performed weekly from 1 month of age onward. Neuroscore was adapted from Nakamura et al.,^40^ providing a combined score of 0 (healthy) to 3 (completely lacking) of the forelimb and hindlimb flexion upon suspension by the tail. At 7 months of age, mice were tested on various motor skill assays, as described previously by Dooves et al.^9^^,^^33^ In brief, the latency of traversing a narrow beam was timed and averaged over three sessions. Accuracy was additionally measured during these sessions by counting the number of missteps animals made while traversing. Grip strength of the front and hind limbs combined was recorded as animals held on to a metal grid (Colin Instruments); scores were averages of five measurements conducted sequentially. Experiments were conducted in procedure rooms and placed back in their home cages in the housing facility for a minimum of 2 h between experiments. No confounding variables were recognized to be controlled for. Animals were tested by researchers blind to the experimental condition.

### Immunohistochemistry

For immunohistochemistry, slides were washed in PBS three times in 10 min. Slides then received heat-mediated antigen retrieval treatment in citrate buffer (0.01 M, pH 6; 0.1 M sodium citrate, 0.1 M citric acid in MQ H_2_O) at 90°C and were left to cool down for 40 min. Slides were rinsed once in PBS and incubated with blocking buffer (5% normal goat serum, 0.1% BSA, 0.3% Triton X) for 1 h at room temperature (RT). Tissue was then incubated with primary antibodies (GFP [1:1,000, Aves Labs, GFP-1020] and/or HA [1:1,000, Abcam, Ab130275]) diluted in blocking buffer for another hour at RT prior to overnight incubation at 4°C. The next day, slides were washed in PBS and subsequently incubated in secondary antibodies diluted in blocking buffer for 2 h at RT. Slides were washed in PBS three times for 10 min once more and finally incubated with 4′,6-diamidino-2-phenylindole (DAPI, 300 nM) for 2 min. After a final wash in PBS, slides were covered with Fluoromount (Southern Biotech, AL, USA) and cover slips and stored at 4°C.

### H&E histology

H&E histology was performed to analyze tissue morphology. Microscopy slides with tissue sections were washed 6 times for 5 min in PBS to remove OCT. Tissue was then briefly rinsed in MilliQ H_2_O before staining with hematoxylin (Sigma) for 1.5 min. Slides were then rinsed under running tap water and differentiated in 1% acid alcohol (HCl in 70% EtOH) for 30 sec. Slides were rinsed once more in running tap water and subsequently blued in 0.1% sodium bicarbonate (in MilliQ H_2_O). Slides were stained with eosin Y (Sigma) for 1 min, rinsed in running tap water, kept in MilliQ for 10 min, and rinsed once more under running tap water. Slides were dehydrated in (30%, 70%, and 96%, and 100%) EtOH, cleared in Xylene, and mounted in Depex (Serva).

### AAV serotype assessment

To compare the tropism and spread of three different AAV serotypes, a CMV-GFP construct (hCMV-chI-EGFP-WPRE-SV40p(A)) was used to produce AAV2/5, AAV2/8, and AAV2/9 virus as described above with titers of 1.4 × 10^13^ vector genomes (vg)/mL, 1.3 × 10^13^ vg/mL, 5.2 × 10^12^ vg/mL, respectively. ICV injections of 2 × 10^10^ vg of virus per animal were performed as described in the section “ICV injections” in the “materials and methods” section. Brains were collected at 5 or 8 weeks, or 9 months of age; spinal cords were additionally collected at 9 months of age. Immunohistochemistry was performed after tissue processing using NeuN (1:500, Millipore, MAB377), GFAP (1:1,000, DAKO, Z0334), and Olig2 (1:1,000, Millipore, AB9610) to determine viral tropism.

### Cell counts

Slices were photographed with a Leica DM6000B microscope (Leica Microsystems). Cell counts were performed in ImageJ by a researcher blind to the genotype and the treatment of analyzed animals. The number of GFP^+^ cells were determined in the cortex, ventral areas, olfactory bulb, cerebellum, hippocampus, white matter tracts (corpus callosum and fornix), and brainstem in animals treated with serotype AAV2/5, 2/8, or 2/9.

### Statistics

Statistical tests to be used were determined *a priori* and performed in SPSS 26.0. Normality of the data was checked by Shapiro-Wilk test. If test assumptions were met, independent-samples t tests or one-way ANOVAs were performed to compare groups. Tukey's b was used as a *post hoc*. In case of non-homogeneity of variances between groups in an ANOVA, determined with a Levene's test, a Welch test with Games-Howell *post hoc* was used. In case of non-parametric data, Mann-Whitney U tests or Kruskal-Wallis tests were performed.

## Supplemental information

Document S1. Figure S1 and Table S1

Document S2. Article plus supplemental information
