# Supplementary material for: In vivo targeting of a variant causing vanishing white matter using CRISPR/Cas9
Source: Mol Ther Methods Clin Dev. 2022 Feb 23;25:17–25. doi: 10.1016/j.omtm.2022.02.006 (PMC8917273; doi:10.1016/j.omtm.2022.02.006)
Supplement: Document S1. Figure S1 and Table S1 [file mmc1.pdf]

OMTM, Volume *25*

Supplemental information

In vivo targeting of a variant causing vanishing white matter using CRISPR/Cas9

Anne E.J. Hillen, Martina Hruzova, Tanja Rothgangl, Marjolein Breur, Marianna Bugiani, Marjo S. van der Knaap, Gerald Schwank, and Vivi M. Heine

Supplementary information

Materials & methods

Table S1. Number of animals used per assay and statistical descriptors

**Assay**	**N**	**M + SD (or median + range)**	**95% confidence interval & effect size**
Serotype pilot spread<br><br>Total n=7<br>If n=1, 2 slices were analyzed per animal	**AAV2/5**:<br>5 weeks n=1 +<br>2 months n=1	Cortex: M=32.3, SD=1.39<br>Ventral areas: M=9.7, SD=0.07<br>Olf. bulb: M=47.7, SD=6.32<br>Cerebellum: M=2.4, SD=0.39<br>Hippocampus: M=5.4, SD=0.37<br>White matter: M=0.7, SD=0.31<br>Brain stem: M=3.0, SD=2.58	N/A
	**AAV2/8**:<br>5 weeks n=2 +<br>2 months n=1	Cortex: M=42.2, SD=4.65<br>Ventral areas: M=11.0, SD=3.68<br>Olf. bulb: M=37.6, SD=6.80<br>Cerebellum: M=3.3, SD=1.15<br>Hippocampus: M=6.5, SD=N/A<br>White matter: M=2.2, SD=0.78<br>Brain stem: M=2.1, SD=1.69	
	**AAV2/9**:<br>5 weeks n=1 +<br>2 months n=1	Cortex: M=51.8, SD=5.49<br>Ventral areas: M=15.5, SD=15.21<br>Olf. bulb: M=24.3, SD=4.85<br>Cerebellum: M=3.5, SD=0.13<br>Hippocampus: M=9.1, SD=3.89<br>White matter: M=0.1, SD=0.14<br>Brain stem: M=7.4, SD=N/A	
Serotype pilot astrocytic tropism<br><br>Total n=7<br>If n=1, 2 slices were analyzed per animal	**AAV2/5**: 5 weeks n=1 + 2 months n=1	Grey matter: M=30.8, SD=4.47<br>White matter: M=97.2, SD=3.93	N/A
	**AAV2/8**: 5 weeks n=2 + 2 months n=1	Grey matter: M=42.8, SD=6.39<br>White matter: M=82.4, SD=5.18	
	**AAV2/9**: 5 weeks n=1 + 2 months n=1	Grey matter: M=26.4, SD=4.23<br>White matter: M=34.3, SD=39.83	
Balance beam latency<br><br>Total n=79	**CRISPR^{hom}: n=10**<br>**CRISPR^{het}: n=24**<br>**Control^{hom}: n=21**<br>**Control^{het}: n=24**	M=23.2, SD=9.29<br>M=12.3, SD=6.41<br>M=18.3, SD=8.08<br>M=4.5, SD=1.45	16.60-29,88<br>9.55-24.97<br>14.59+21.95<br>3.84+5.06<br>η^2 = .525
Balance beam accuracy<br><br>Total n=79	**CRISPR^{hom}: n=10**<br>**CRISPR^{het}: n=24**<br>**Control^{hom}: n=21**<br>**Control^{het}: n=24**	M=11.9, SD=4.81<br>M=4.9, SD=3.41<br>M=18.3, SD=8.08<br>M=4.5, SD=1.45	8.42-15.30<br>3.43-6.31<br>14.59-21.95

			3.84-5.06<br>η^2 = .690
Grip strength<br><br>Total n=80	**CRISPR^{hom}: n=10**<br>**CRISPR^{het}: n=24**<br>**Control^{hom}: n=21**<br>**Control^{het}: n=25**	M=1.4, SD=0.23<br>M=1.7, SD=0.22<br>M=1.6, SD=0.19<br>M=1.9, SD=0.18	1.28-1.61<br>1.63-1.81<br>1.54-1.71<br>1.87-2.02<br>η^2 = .422
Next Generation Sequencing- NHEJ<br><br>Total n=97	**CRISPR: n=51**<br>**CRISPR^{hom}: n=21**<br>**CRISPR^{het}: n=30**<br>**Control: n=46**<br>**Control^{hom}: n=23**<br>**Control^{het}: n=23**	M=4.6, SD=1.72<br>M=4.9, SD=1.92<br>M=4.5, SD=1.55<br>M=0.08, SD=0.05<br>M=0.05, SD=0.05<br>M=0.08, SD=0.05	4.16-5.12<br>4.01-5.76<br>3.89-5.05<br>0.07-0.10<br>0.07-0.10<br>0.06-0.10<br>CRISPR vs. controls: Cohens's d =3.792
Next Generation Sequencing- HDR<br><br>Total n=97	**CRISPR: n=51**<br>**(CRISPR^{hom}: n=21**<br>**CRISPR^{het}: n=30)**<br>**Control: n=46**<br>**(Control^{hom}: n=23**<br>**Control^{het}: n=23)**	M=0.3, SD= 0.17<br>M=0.3, SD=0.20<br>M=0.2, SD=0.13<br>M=0.004, SD=0.01<br>M=0.1, SD=0.05<br>M=0.08, SD=0.05	0.22-0.31<br>0.24-0.42<br>0.17-0.27<br>0.001-0.006<br>0.07-0.10<br>0.06-0.10<br>CRISPR vs. controls: η^2 = .778
Weight at 42 days old<br><br>Total n=149	**CRISPR^{hom}: n=30**<br>**CRISPR^{het}: n=42**<br>**Control^{hom}: n=40**<br>**Control^{het}: n=37**	M=13.7, SD=2.45<br>M=16.4, SD=3.31<br>M=15.4, SD=2.50<br>M=15.6, SD=2.48	12.30-15.00<br>15.26-17.57<br>14.50-16.33<br>14.75-16.43<br>CRISPRhom vs. CRISPRhet: η^2 = .852
Weight at 7 months old<br><br>Total n=80	**CRISPR^{hom}: n=10**<br>**CRISPR^{het}: n=24**<br>**Control^{hom}: n=21**<br>**Control^{het}: n=25**	M=19.6, SD=2.80<br>M=28.0, SD=7.08<br>M=23.6, SD=3.90<br>M=32.5, SD=5.11	17.60-21.60<br>24.98-30.96<br>21.87-25.42<br>30.35-24.56<br>η^2 = .482
Neuroscore at 7 months old<br><br>Total n=80	**CRISPR^{hom}: n=10**<br>**CRISPR^{het}: n=24**<br>**Control^{hom}: n=21**<br>**Control^{het}: n=25**	M=0.60, SD=0.46<br>M=0.63, SD=0.58<br>M=0.48, SD=0.33<br>M=0.06, SD=0.22	0.27-0.93<br>0.38-0.87<br>0.32-0.63<br>-0.03-0.15<br>η^2 = .264
H&E<br><br>Total n=10	**CRISPR^{hom}:**<br>34-44 days old n=4<br>9 months old n=1<br>**CRISPR^{het}:**<br>34-44 days old n=1<br>9 months old n=1<br>**Control^{hom}:**<br>9 months old n=2<br>**Control^{het}:**<br>9 months old n=1	N/A	N/A

Timed culling of animals	12 weeks old<br>**Control**^{hom} n=4<br>**Control**^{het} n=4
	5 months old<br>**CRISPR**^{het} n= 4<br>**Control**^{hom} n= 15<br>**Control**^{het} n=8
	8 months old<br>**CRISPR**^{het} n=12<br>**Control**^{hom} n=9<br>**Control**^{het} n=12
	9 months old<br>**CRISPR**^{hom} n=10<br>**CRISPR**^{het} n=13<br>**Control**^{hom} n=12<br>**Control**^{het} n= 13

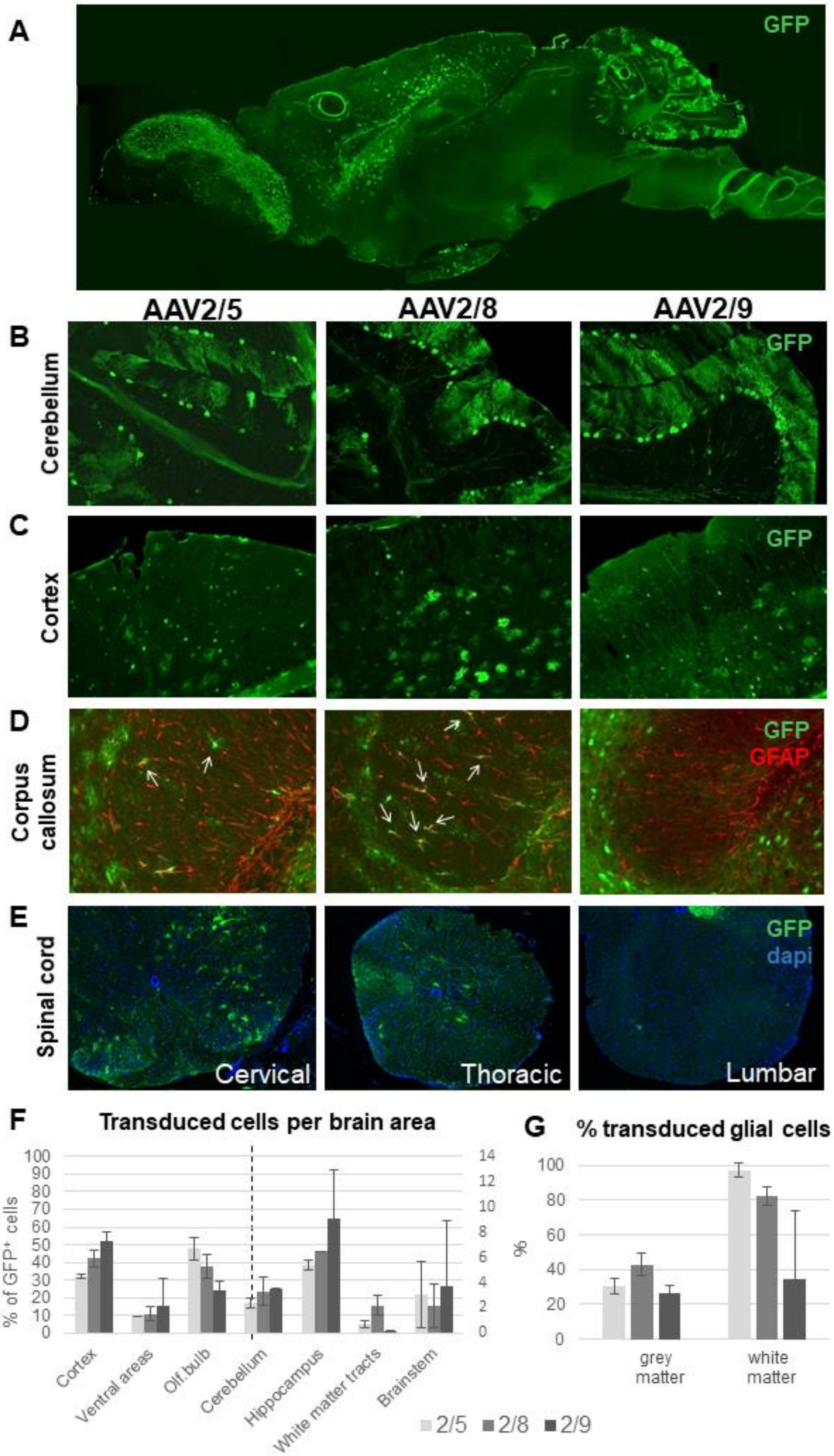

Supplemental Figure 1. **AAV serotypes were tested for astrocytic transduction *in vivo***. (**A**) Neonatal mice received intracerebroventricular injections of AAV, which

resulted in a wide spread of the virus at 5 weeks of age. Expression of CMV-GFP (green), conferred by AAV with chimeric serotypes AAV2/5, AAV2/8, or AAV2/9, was seen in the (**B**) cerebellum and (**C**) cortex. (**D**) In the corpus callosum at 5 weeks of age, co-localization of GFP^+ cells with GFAP (red) is indicated by arrows. (**E**) The spinal cord showed transduced cells at the cervical, thoracic and lumbar portions at 9 months post-injection of AAV2/8. (**F**) The number of transduced cells per region are quantified (dashed line assigns regions to the two different y-axes), and (**G**) expressed in the percentage of glial cells of the total number of transduced cells. n=2 animals for AAV2/5 and AAV2/9, and n=3 animals for AAV2/8 until 5 or 8 weeks of age. n=24 for AAV2/8 until 9 months of age.
